# Non-Specific Adsorption Reduction Methods in Biosensing

**DOI:** 10.3390/s19112488

**Published:** 2019-05-31

**Authors:** Jessanne Y. Lichtenberg, Yue Ling, Seunghyun Kim

**Affiliations:** 1 Department of Electrical and Computer Engineering, School of Engineering, Baylor University, Waco, TX 76798, USA; jess_lichtenberg1@baylor.edu; 2 Department of Mechanical Engineering, School of Engineering, Baylor University, Waco, TX 76798, USA; stanley_ling@baylor.edu; 3 Department of Electrical and Computer Engineering, School of Engineering, Baylor University, Waco, TX 76798, USA

**Keywords:** biosensors, non-specific adsorption, biofouling, surface functionalization, proteins

## Abstract

Non-specific adsorption (NSA) is a persistent problem that negatively affects biosensors, decreasing sensitivity, specificity, and reproducibility. Passive and active removal methods exist to remedy this issue, by coating the surface or generating surface forces to shear away weakly adhered biomolecules, respectively. However, many surface coatings are not compatible or effective for sensing, and thus active removal methods have been developed to combat this phenomenon. This review aims to provide an overview of methods of NSA reduction in biosensing, focusing on the shift from passive methods to active methods in the past decade. Attention is focused on protein NSA, due to their common use in biosensing for biomarker diagnostics. To our knowledge, this is the first review to comprehensively discuss active NSA removal methods. Lastly, the challenges and future perspectives of NSA reduction in biosensing are discussed.

## 1. Introduction

Creating sensitive, selective, and stable biosensors for the timely identification of disease biomarkers is greatly important [1]. In recent years, copious research has been focused on the development of new methods for early disease detection [1]. Numerous in-depth reviews on biosensors exist for readers of interest [2,3,4,5]. The goal for a biosensor is to achieve the maximum sensitivity, specificity and selectivity for a particular application [6]. In order to accomplish this, both the sensor element and the neighboring surfaces have to be unreactive to non-specific adsorption [6]. This is imperative for limiting the attachment of target molecules to the sensitive areas of the sensor by subsequent specific functionalization [6]. Especially for small-scale sensors, it is crucial to accomplish controlled patterning of functional molecules. This is because the size of the molecules used for passivation and capture, as well as the analytes of interest, have similar dimensions to the sensor element or at least comparable to the size of the sensitive area [6]. 

Surface-based sensing is a common method of biosensing, exemplified by immunosensors (e.g., ELISA and SPR), microfluidic biosensors, and electrochemical biosensors. A model biosensor contains functional areas coinciding with the sensing area and inactive areas everywhere else to promote minimum sample consumption [6]. However, biomolecular areas of surface-based sensors often come into contact with complex mixtures of proteins and other molecules during their use [7]. Most surfaces are particularly prone to non-specific and irreversible adsorption of proteins, known as non-specific adsorption (NSA) [7,8,9,10]. NSA happens when a molecule adsorbs to a sensor’s surface, resulting in high background signals that are indiscernible from the specific binding [9]. This phenomenon occurs because of physisorption and can decrease the sensor’s performance [1,9].

It is important to note that for some sensors, NSA can be distinguished from the analyte signal. This is the case for biodetection with attenuated total internal reflection Fourier transform infrared (ATR-FTIR) or ellipsometry. ATR-FTIR spectroscopy have been used for biotin detection, where the specific binding was able to be differentiated from the non-specific protein binding [11]. Ellipsometry, which measures the change of polarization upon reflection or transmission, has been used to study in vitro polyclonal antibody adsorption [12]. This technique can be used to identify the specifically adsorbed proteins from a complex solution. However, this method is not successful for monoclonal antibodies, and requires an optically reflective, flat surface. Infrared (IR) ellipsometry has been demonstrated as a label-free imaging method to map different materials on a biochip [13]. Compared to conventional chemiluminescence imaging, this method was found to be able to map larger sample areas with greater resolution (300 μm × 300 μm), attained by using radiation from an IR synchrotron beamline. While imaging techniques are useful for discriminating between specific and non-specific adsorption, their implantation is not applicable to all sensors. A majority of surface-based sensors suffer from the effects of NSA, particularly microfluidic biosensors.

Microfluidics is the field of fluid handling technology involving very small volumes of liquids in the range of 10^−6^ to 10^−18^ L [4]. Microfluidics have numerous applications including biomedicine, cell analysis, drug screening, cell biology, and biosensing [4]. In recent years, momentous work has been done to reduce the size of conventional biosensors using microfluidics for lab-on-a-chip practices [8]. Numerous benefits exist for using microfluidics such as the low consumption of costly reagents, minimal handling of hazardous materials, short reaction time, multiple sample detection, increased portability and design versatility [8]. Decreasing the sensor dimensions also enhances signal-to-noise ratio by increasing the signal density and reducing background signals [14]. Literature on the design and applications of microfluidic biosensors is vast [5,15,16,17,18]. Microfluidic biosensors often have immobilized bioreceptors, such as antibodies [19], enzymes [20], DNA [21], and linker molecules such as SAMs (self-assembled monolayers) to improve surface immobilization. However, the linker molecules are prone to NSA, resulting in decreasing sensitivity and false responses [8].

The interaction between the bioreceptor and analyte can be categorized into affinity or catalytic [4]. Affinity biosensors are based on the binding affinity between the analyte and bioreceptor (e.g., antibody-antigen). In catalytic biosensors a chemical reaction produces a product which can be correlated with the concentration of target analyte [4]. An example of a catalytic sensor is a glucose biosensor which uses the enzyme glucose oxidase that catalyzes the oxidation of glucose to hydrogen peroxide. This review focuses more on the NSA of affinity biosensors due to their prevalence in diagnostic biomarker protein detection.

The balance between simultaneous sensing and NSA removal is a key obstacle in biosensing [22]. Numerous methods exist to reduce non-specific interactions on sensing surfaces. Blocking methods aim to reduce NSA by coating the surface, while removal methods generate surface shear forces to overpower the adhesive forces of the non-specifically adsorbed molecules [9]. Physical removal methods use a transducer, and are typically categorized as electromechanical or acoustic devices. Another less commonly used technique is hydrodynamic removal, which relies solely on the fluid flow to generate the shear forces. 

## 2. Non-Specific Adsorption

**Figure 1:**
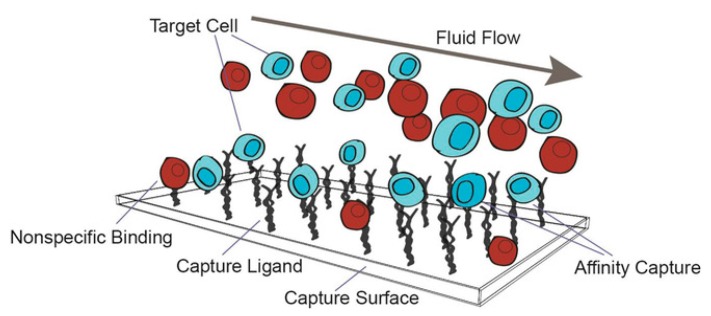
Target molecules (blue) in a sample containing background molecules (red) flow through a microchannel. The surface is covered with bioreceptors. Affinity capture is when the receptors on the target molecule are recognized by the capture ligand. Non-specific binding occurs when a background molecule binds to the ligand or the channel surface without forming ligand-receptor bonds. Adapted with permission from ref [24]. Copyright 2018 John Wiley and Sons.

Adsorption is the adhesion of atoms, ions or molecules from a gas, liquid or dissolved solid to a surface [23]. There are two types of adsorption: physical and chemical. Physical adsorption, or physisorption, results from intermolecular forces including hydrophobic forces, ionic interactions, van der Waals forces, and hydrogen bonding. Chemical adsorption, or chemisorption, results from chemical interactions such as covalent binding. In NSA (Figure 1), molecules are adsorbed to a surface through the weaker adsorption, physisorption [9].

Non-specific adsorption (NSA), also known as non-specific binding or biofouling is a persistent challenge in biosensing [5,8,25,26,27]. While NSA is an issue in other biological fields including implantable biomedical devices [28,29,30], and marine equipment [31,32], this report will focus on NSA for biosensing applications. Most biomolecular surfaces, whether comprised of antibodies, enzymes or proteins, experience the common issue of hindrance from non-specific species [25]. For biosensors, the molecules typically adsorb to the sensing surface from a liquid medium. 

Immunosensors, a common type of biosensor, use antibodies or antigens as the bioreceptor. For antibodies, there are two types of non-specific adsorption: immunological and methodological. Immunological has more to do with the affinity between the antibody and antigens, and cannot be improved without using different proteins. Methodological non-specificity can occur due to a variety of reasons, but is usually a combination of protein-protein interactions, surface protein denaturation or mis-orientation, substrate stickiness, non-specific electrostatic binding to charged surface, and adsorption of molecules in free spaces [25]. This phenomenon can result in four different types of NSA: (1) molecules adsorbed on vacant spaces (2) molecules adsorbed on non-immunological sites (3) molecules adsorbed on immunological sites, allowing access to antigens and (4) molecules adsorbed on immunological sites. Non-specific adsorption leads to elevated background signals that cannot be discriminated from specific binding [9]. These false-positive signals affect the dynamic range [25], limit of detection [2,9], reproducibility, selectivity, and sensitivity [9]. The reduction of NSA is crucial in the development of biosensors, especially for point-of-care clinical diagnostics [5]. 

Limiting NSA has been investigated for decades [33]. Recent reviews focus on NSA reduction for certain sensor types such as the suppression of protein NSA in the development of electrochemical immunosensors [26], antifouling materials for SPR sensors [34], nanoscale sensors [6], and whispering gallery mode optical biosensors [35]. With the increased trend in micro/nano-scale biosensors, a shift from passive methods to active removal methods is noticed in literature, which is the focus of this review.

## 3. Methods of NSA Reduction

**Figure 2:**
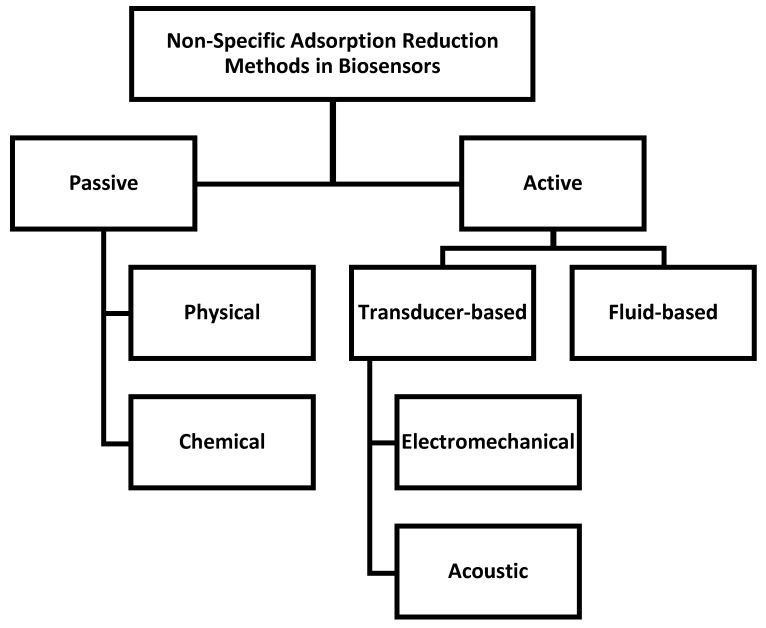
Chart outlining the main mechanisms of NSA reduction (passive and active) and sub-categories of each.

Non-specific adsorption can be reduced by a number of approaches with varying complexity [7]. NSA reduction methods can be generally divided into two categories: passive methods and active methods (Figure 2). The passive methods aim to prevent undesired adsorption by coating the surface, and can broadly be sub-categorized as physical and chemical. These methods have existed for several decades and are vast in the literature. In contrast, active methods dynamically remove adsorption post-functionalization and are a more recent technique. Active removal methods can be further categorized as transducer-based or fluid-based. The transducers used are typically electromechanical or acoustic. Fluid-based methods utilize the pressure-driven flow of microfluidics to shear away biomolecules. This review will briefly describe the passive methods, which have been thoroughly described in the literature already [8,25,26,31,36], while focusing on the active methods which have yet to be thoroughly investigated.

### 3.1. Passive Methods

Blocking methods can be categorized into two broad categories: physical (i.e., protein blockers) and chemical (i.e., linker molecules). A recent review has provided an in-depth review of physical and chemical surface modification methods [6]. Therefore, here we will only briefly summarize the main mechanisms in this review and note key examples. Passive methods aim to create a thin hydrophilic and non-charged boundary layer to thwart protein adsorption [9]. The goal of anti-fouling coatings is to minimize the intermolecular forces and interactions between the adsorbing molecules and the host substrate so the molecules can be easily detached and released under low shear stresses, like washing [37]. Materials for non-fouling coatings are usually neutral or weakly negative and well hydrated [36].

#### 3.1.1. Physical

Physical surface modification does not typically change the chemical composition of the surface. The most popular and easiest method to prevent NSA is to use blocker proteins that adsorb to surfaces. For example, serum albumins (e.g., BSA) [38,39], casein [39,40], and other milk proteins [36] are commonly used as blocking agents for enzyme-linked immunosorbent assay (ELISA), Western blotting, and other enzyme based assays [39,41,42]. The underlying assumption of these methods is the idea that a controlled patterning of the surface-active protein will inhibit the immobilization of other molecules to the substrate [7]. Commonly used bovine serum albumin (BSA), protein derived from cows, is easily accessible and inexpensive [38]. However, the usage of BSA often leads to a non-uniform layer on the surface, and studies have reported proteins can still adsorb to a BSA layer [7]. Additionally, while protein blockers can be applied to a variety of surface materials, they can have high lot-to-lot variability, cross-reactivity [43] and alter the original surface properties [44].

For catalytic biosensors, a permselective membrane, with preferential permeation of certain ionic species through ion-exchange membranes, can be used to reduce NSA [14,45,46]. In an amperometric glucose sensor, the ion permselectivity avoids interfaces of the detection of hydrogen peroxide by ascorbate, urate and acetaminophen [14,47]. Additionally, the use of a permselective membrane can reduce the influence of buffer concentration on the sensor signal [48]. Another method of dealing with protein NSA utilizes the Vroman effect [14,49,50]. This is when a weak affinity protein bound to the surface is displaced by a strong affinity protein [17]. The reverse operation does not occur. This phenomenon is due to the fact that protein adsorption capability is largely dependent on the molecular weight [51]. Therefore, in general, a high molecular weight protein adsorbs more strongly than a low molecular weight protein. The displacement of these proteins can be measured by SPR [52].

#### 3.1.2. Chemical

Chemical methods of anti-fouling include using a poly(ethylene glycol) (PEG)/oligo(ethylene glycol) (OEG) based coating [9,37,53,54], functionalized self-assembled monolayers (SAMS) [8,9,55,56,57,58], or zwitterionic polymers [1,9,36,59,60,61]. PEG is a non-ionic, water soluble polymer commonly used to form protein-resistant layers [54]. PEG and OEG have been widely used antifouling materials due to their surface hydration [1], and weakly basic ether linkages [37]. However, they are vulnerable under oxidative conditions, which limits their efficacy for long-term applications [1,54]. Additionally, the auto-oxidation of the terminal hydroxyl group in PEG into aldehydes can deteriorate the specific proteins used for sensing [62]. Some types of PEG are only useful for negatively-charged surfaces, and are most successful with metal oxide surfaces [7]. These compounds are better suited for single proteins, but most often fail in complex mixtures of proteins (e.g., blood plasma [63,64]), or under in vivo conditions [65]. Furthermore, the density and chain length of PEG need to be optimized for minimizing non-specific interactions [53]. The ability of PEG to prevent NSA depends on other parameters, such as temperature [64], and can be unstable [66]. Researchers have used nanostructured surface coatings to reduce protein adsorption as an alternative to polymer modification [67].

Self-assembled monolayers (SAMs) offer an oligoethylene glycol functional group at the solid-water interface [7]. Alkanethiol SAMs are one of the most popularly used bioreceptor linker molecules, due to the two major advantages: plentiful functional groups to immobilize bioreceptors and a dense monolayer that allows little NSA [8]. Sensing surfaces with alkanethiol SAMs suffer from NSA due to three main reasons: polycrystalline gold grain structure, imperfectly formed monolayers, and non-immobilized bioreceptors [8]. Additionally, factors such as surface roughness, annealing, and the use of short or long chain SAMs can affect the results [8]. Furthermore, oligoethylene glycol-terminated SAMs are only compatible with gold and silver surfaces [7], which limits their use considering the variety of substrates used today in biosensing [7,62].

Zwitterion polymers or polyzwitterions are polymers made up of zwitterions, molecules which have both a positive and negative charge [61]. Zwitterion polymers have the same number of anionic and cationic groups, so the overall charge is zero under normal conditions. These ionic groups repel unwanted adhesion with a strong hydration layer through ionic solvation [1,37] and are functional over a large pH window [61]. Zwitterionic polymer brushes may be grafted to or grafted from surface. However, most zwitterionic reagents that can be presented onto substrate surfaces for NSA reduction are usually restricted to betaine and phosphorylcholine related chemicals, whose synthetic processes are slow and difficult [1]. A promising subclass of zwitterionic polymers is polyampholyte polymers, composed of mixtures of charged monomer subunits, whose mechanical properties of the polymers can be tailored. Surface grafted polyampholyte polymer brushes have been demonstrated as an anti-fouling coating for sensing applications in complex media [68].

Challenges with chemical methods include laborious functionalization processes, long-term chemical stability, raising the sensor background signal [9], and the prospect to damage active surface [9,62]. Additionally, some chemical methods have limitations in transducer interfaces as previously described [7,9]. The prevention of NSA with passivation methods often involves the use of harsh chemicals that are normally not appropriate for many biological uses [7].

Manipulating surface chemistry is only part of controlling protein adsorption. The surface topography is key in determining the structure of bound proteins [69] and can be used to control specific protein adsorption [70]. Alternative methods to control NSA is through the patterning or imprinting the surface with a unique surface topography or synthetic polymer. Shi et al. reported a method of imprinting surfaces with nanocavities to specifically recognize template proteins [70]. The authors suggest non-template proteins will be less likely to attach to the imprinted sites because they lack the ability to interlock in the cavities and form a strong bond. The imprinted surface topography was evaluated using tapping-mode atomic force microscopy (AFM). They evaluated three imprinted blood proteins (BSA, IgG, and fibrinogen) compared to a flat control surface, and found a higher number of proteins were adsorbed on the imprints. After using a detergent to clean the surface, the researchers found the protein imprint retained more of the proteins than the control surface, implying a greater affinity at the imprinted cavities. Specific binding sites can be limited to the nanocavities, while elsewhere is blocked with BSA passivation. Roach et al. investigated the effect of surface topography on the binding of proteins [69]. They found differences in how certain protein geometries were affected by surface structure. For example, a globular protein like albumin, is more compatible with a surface with high curvature. However, a rod-like protein such as fibrinogen, is distorted by a curved surface, which promotes structure loss. These effects are important when considering how to optimize specific adsorption and prevent NSA [69].

A synthetic recognition system can also be implemented as an alternative to the typical biological receptor used in sensing [71], such as molecularly imprinted polymers (MIPs). This synthetic recognition system is inexpensive, easy to implement, and allows target species to rebind to the imprinted sites. However, challenges exist the uncontrolled random polymerization produces uneven imprinted sites. Additionally, the use of MIPs is limited to chemical detection or bioassays due to the inability to generate signal output at the surface of the transducer. Molecularly imprinted nanomaterials, such as nanowires, nanotubes, nanofibers have been investigated for chemosensors as a solution to the challenges faced by traditionally imprinted materials. While improvement in achieving sensitive detection has been achieved with these methods, there are still limitations to this method including the need to enhance the specific binding, reduce NSA, and allow for multiplexing and easy integration [71]. Due to these limitations, active methods have emerged as an alternative to combat NSA.

### 3.2. Active Methods

Methods of active NSA removal have emerged in the past decade as a promising approach to reduce NSA in biosensors. These methods produce surface shear forces, which are stronger than the adhesive force of the weakly bound NSA molecules. Meanwhile, the specific proteins are not removed because the affinity of specific proteins toward a ligand is several orders of magnitude larger than that of non-specific proteins [22,72]. Transducer-based approaches can be used for numerous types of proteins, making them time-saving, easy operating, and efficient methods for removing NSA [9]. Transducer devices to remove NSA can be categorized as electromechanical or acoustic (Figure 2) [9]. A summary table of active NSA removal methods is provided in Table 1.

#### 3.2.1. Transducer-Based

The shearing forces in electromechanical methods are generated from the electrical or mechanical transducers. Some examples of electromechanical methods include ac-EHD (alternating current electrohydrodynamics) induced nanoshearing [10,73,74,75,76], hypersonic resonance [9], and resonant cantilever vibration [44].

**Figure 3:**
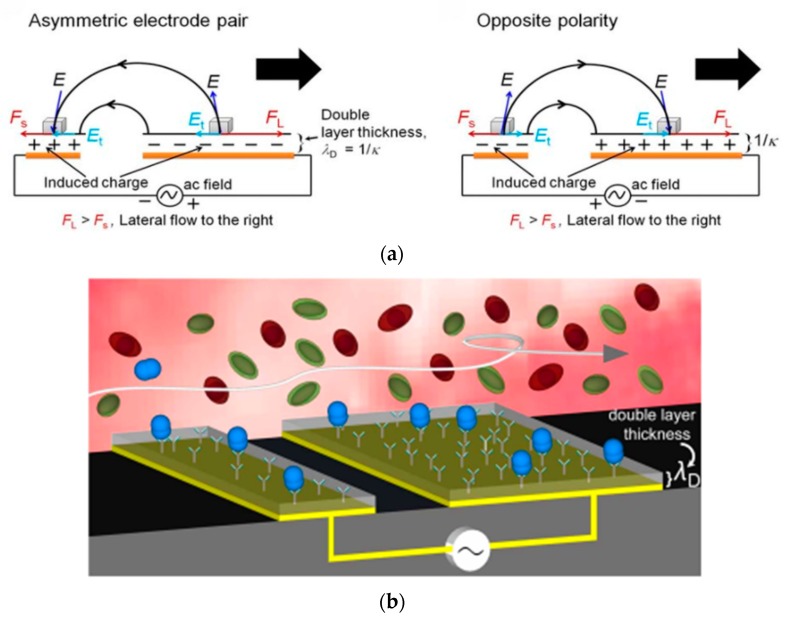
Ac-EHD induced fluid flow (**a**) Mechanism of how the electric field induces charges in the double layer of each electrode experience. Adapted with permission from [70]. Copyright 2014 Springer Nature. (**b**) Schematic of ac-EHD induced nanoshearing. The target proteins (blue) are bound to the bioreceptors while the non-specifically bound molecules (green and red) are removed. Adapted with permission from [10]. Copyright 2015 Springer Nature.

A sensor has been developed that uses alternating current electrohydynamics (ac-EHD) induced surface shear forces, or nanoshearing, to improve the analyte capture of the sensor and remove weakly (non-specifically) bound molecules from the electrode surface [10]. The working principle is that the ac-EHD induced flow generates shearing forces within nanometer distances of the electrode surface to produce lateral fluid flow (Figure 3a). This not only mixes the fluid around the sensor surface to enhance capture efficiency through increased sensor-target collisions, but the ac-electric field can enable selection of specifically bound proteins over non-specifically bound proteins (Figure 3b). This produced 100 fg·mL^−1^ naked eye detection of multiple protein targets spiked in human serum [10]. The results were compared to that of a pressure-driven flow system with the same flow rate. The authors found the absorbance measurements were 1000 times better for the ac-EHD system than the hydrodynamic. Additionally, they noted limitations of pressure-driven flow including the stationary boundary layer of fluid, which does not allow for surface shear force manipulation, and the external syringe pump needed that is not easily integrated into portable devices. This system is multiplexible via several individual channels for distinct functionalization of different bioreceptors. This device has been applied for sensing and biofouling of exosomes [75] as well as being incorporated with a surface-enhanced Raman scattering (SERS)-based immunoassay [74]. A complete look at ac-EHD use in microsystems has been recently published for the interested reader [84].

**Figure 4:**
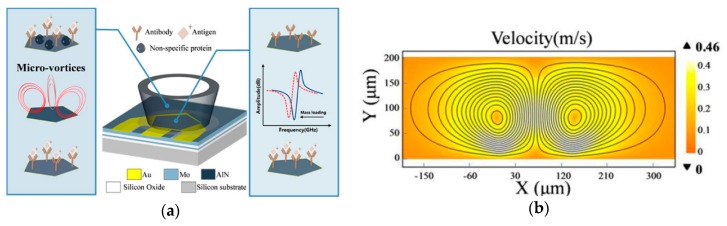
Hypersonic resonator for biofouling removal and protein detection: (**a**) Illustration of microvortexes in liquid triggered by the hypersonic resonator; (**b**) Simulation of the fluid motion triggered by the hypersonic resonator. Adapted with permission from ref [9]. Copyright 2017 the American Chemical Society.

Since the shearing forces typically come from electrical or mechanical vibration of the devices, the NSA removal is limited to the proteins adsorbed on the transducer surface [9]. Pan et al. proposed a solution to this problem by using a microfabricated 2.5 GHz hypersonic resonator to generate microvortexes in the fluid, which results in the attenuation of acoustic energy into the liquid (Figure 4) [9]. The resulting drag and lift forces along the liquid-solid interfaces were used to control the NSA removal. The experimental results supported the mathematical and simulated models. The proteins were modeled as spherical proteins with a diameter of 100 nm, which correlates to a 500-mW power to remove them. However, if the proteins are 1–10 nm, the required power is very high, ~5 W. The mass-sensitive hypersonic resonator displayed a shift in the resonance frequency as each step was performed. However, frequency change cannot differentiate between specific or nonspecific binding. Therefore, fluorescent microscopy was used to verify the NSA removal with a specific immunoglobulin G (IgG) and nonspecific IgG. The quantitative testing showed an 83.8% fluorescence intensity decrease for the nonspecific binding sites after hypersonic treatment and only 6.1% with specific binding sites. Additionally, the vertical orientation of the vortexes allows a contactless setup, which enables the resonator to be combined with other surface based biosensors. The detachable actuator enables reuse. The authors determined the drag force was the primary force in removing NSA. 

Johnson and Mutharasan demonstrated that transverse modes in electrochemical piezoelectric-excited millimeter-sized cantilevers (ePEMCs) caused the release of adsorbed bovine and human serum albumin proteins from sensor surfaces [44]. They first determined ePEMC transverse mode vibration does cause streaming and tested different excitations voltages and particle concentrations. Then, they tested the release of BSA and human serum albumin (HSA), and repeated the experiments with thiolated single-stranded DNA strands to Au sites, which has a 4 times higher binding energy than BSA. Videos of the experiments were recorded to calculate the streaming velocities and visualize the ePEMC vibration-induced flow. The results were determined through electrochemical and mass change sensing techniques, and further verified with wet chemical-based spectroscopic analysis. The study found the release of the NSA proteins was caused by a combination of surface strain energy, body forces, and acoustic streaming associated with hydrodynamic effects. Furthermore, this work could be applied to removing specific binding for bioseparation and diagnostic applications. Before this work, few studies had investigated the non-specific interactions of the non-target species [85]. In recent years, the use of micro cantilever-based sensing systems has increased due to their anticipated high sensitivity [85,86,87,88,89,90,91].

Acoustic wave sensors use acoustic energy to disturb the bonds between the sensing layer and analyte, coercing only the molecules with the higher affinity (i.e., specific proteins) to stay attached. The fluid motion induced from high-intensity sound waves is called acoustic streaming [62,92,93]. The produced sound fields cause tangential fluid motion along the fluid boundaries. The fluid motion applies a steady viscous stress on the boundary layer, causing liquid circulation near the boundaries. These stresses are strong enough to remove loosely bound molecules on the surface of the device. Acoustic streaming enables higher accuracy measurements and reusability of the devices [62]. Examples of acoustic transducer devices include surface acoustic wave (SAW) [62,77,78], orthogonal surface acoustic wave [21], longitudinal acoustic wave (LAW) [80], and bulk acoustic wave [81,82]. Detailed information on acoustic sensing has been recently published [94,95,96,97,98,99].

**Figure 5:**
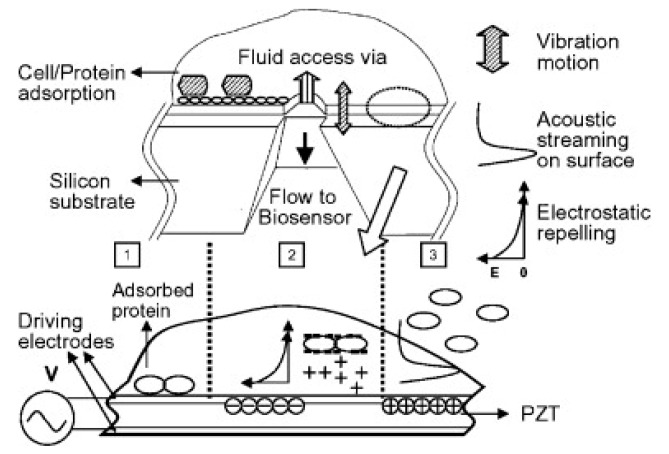
Schematic diagram of the piezoelectric membrane as an implantable sensor coating. The membrane is composed of two driving electrodes with a piezoelectric material in between. Adsorbed proteins can be desorbed by an electric field and carried away by acoustic streaming generated by the vibration. Reprinted from [65] with permission from Elsevier.

Combined electromechanical systems are under investigation too. The nanomolecule desorption mechanism was demonstrated using a combination of electric field and mechanical vibration to remove BSA and IgG from metal coated lead zirconate titanate (PZT) surfaces (Figure 5) [65]. Surface acoustic waves propagating on the surface of a piezoelectric device can be used to induce acoustic streaming within the fluid [62]. Previous studies demonstrated the ability of Rayleigh wave modes to release NSA proteins from the surfaces of SAW devices [78]. The acoustic waves generated from the piezoelectric coupling successfully induced fluid motion (acoustic streaming) and physically forced weakly bound non-specific protein species from their binding sites, improving the biosensor response [78]. The work was furthered by using fluid-solid interaction models to explore parameters for elevating induced acoustic-streaming velocities and forces to eradicate biofouling, while minimizing the effect of streaming-induced removal forces on the antibody sensing layer in immuno-SAW sensors [62]. Singh et al. reported multi-directional transducer-based SAW devices on a single piezoelectric platform produces the benefit of carefully using aspects which are particular to acoustic waves spreading along a given crystallographic orientation [22].

**Figure 6:**
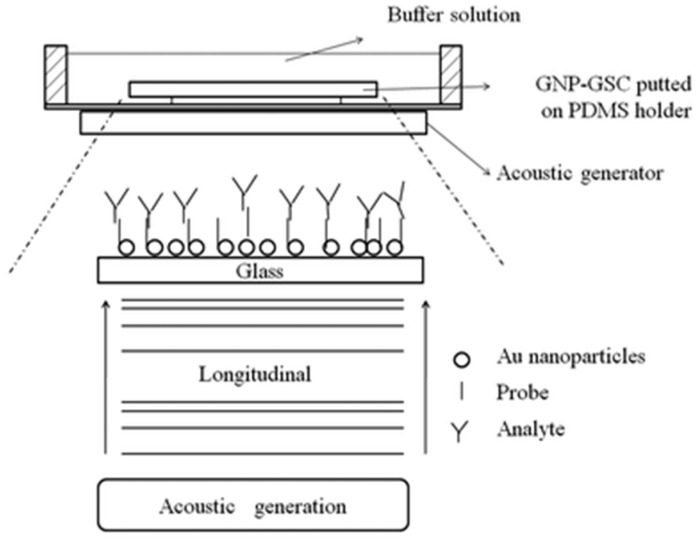
Schematic representation and working principle of the experimental setup for generating LAWs and transmitting to a GNP-GSC through buffer solution. Biomolecular binding at the surface of the functionalized GNPs removed by the acoustic wave effect. Reproduced from [81] with permission from The Royal Society of Chemistry.

Hsu et al. used a piezoelectric transducer to produce varied amplitude and swept-frequency longitudinal acoustic waves remove non-specifically bound biomolecules (Figure 6) [81]. They used a sample chip composed of a diced glass slide chip (GSC) modified with gold nanoparticles (GNPs) and immobilized biotin as a probe. The absorbance spectra of the localized surface plasmon resonance (SPR) band were used for quantitative analysis of the removal efficiency. They demonstrated that even strongly bound streptavidin molecules were able to be removed from the biotin-functionalized GNP-GSC surface [81]. The study also aimed to investigate the regeneration of specific binding sites. The device was found to be reusable at least five times for regeneration, and the NSA removal ability was tested with four different adsorbing molecules: BSA, streptavidin, anti-biotin, and anti-fibrinogen. A range of voltages was tested to optimize removal efficiency for of the non-specific biomolecules. This non-contact system can remove non-specific or specifically bound molecules from the surface. 

#### 3.2.2. Fluid-Based

**Figure 7:**
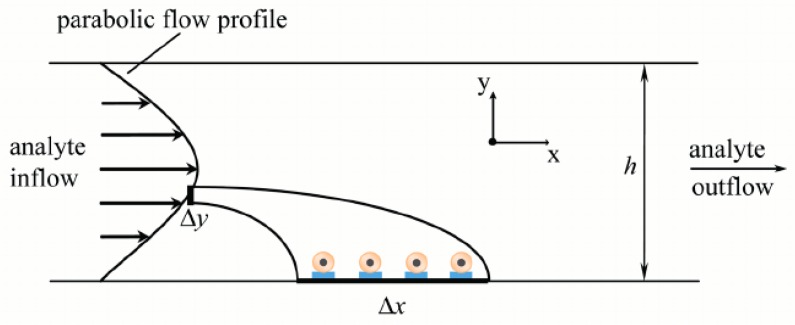
Diagram of how pressure-driven flow creates a parabolic velocity profile, which can behaves on immobilized bioreceptors and bound analytes in microchannel. Reproduced from [104] with permission of AIP Publishing.

Fluid-based methods of NSA removal utilize the pressure-driven flow already integral in microfluidic lab-on-chip systems to remove NSA on the sensing surface along the bottom channel wall (Figure 7). Other applications of fluid manipulation in channels includes the use of hydrodynamic forces to manipulate membrane-bound proteins in a cell membrane [100] and for polystyrene sphere removal on quartz surfaces [101,102]. Another example is using a confining sheath fluid within a microfluidic channel to prevent NSA [103]. However, this method is limited in scope to adsorption-free T-sensor measurements to be made within the core of the flow channel [103] and is not applicable for surface-based sensing.

**Figure 8:**
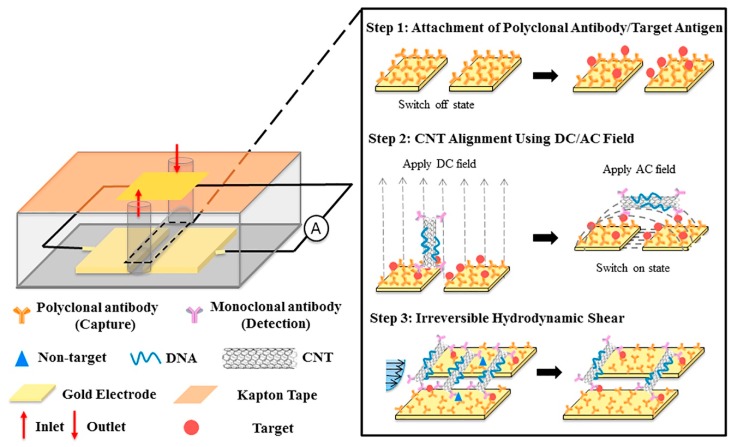
Schematic of CNTs switch sensor for protein detection. Reproduced from [84] with permission of Elsevier.

Hydrodynamic shearing is a transducer-free method of non-specific binding removal, not commonly found in literature. Li et al. applied this technique to effectively remove non-specifically bound carbon nanotubes (CNT) on a multiplexible biosensor after electrophoresis alignment using a 1X PBS solution shearing at 500 µL/min (Figure 8) [84]. The CNTs bridge across the two parallel electrodes to bind with targets. Due to the large hydrodynamic drag imposed by the cross-flow of the cylindrical CNTs with high aspect ratio, a critical hydrodynamic shear rate removed the non-target linkers of the aligned CNTs. Detection limits of 100 aM and 10 fM in pure samples for two ELISA tests: biotin/streptavidin and HER2 (Human epidermal growth factor receptor 2)/HER2 antibody were achieved. For both models, the dynamic range was tuned up to five orders of magnitude by increasing the CNT numbers, with high sensitivity and specificity. The researchers used biotin-streptavidin as the antigen-antibody pair to optimize the flow rate [84]. The key step in this process is the DC electrophoresis and AC dielectrophoresis to align the CNTs before shearing. This requires gold electrodes fabricated into the device and additional time (20 min) for the CNTs assembly. This system is proposed as an improved sensitivity ELISA assay, with a cost estimate of $20 USD per test. 

## 4. Discussion

In summarizing the various methods of NSA reduction in sensing, the main disadvantage of the passive methods is their inconsistency and tedious processes required. The main disadvantage of the transducer methods is that they require extra and equipment. The main disadvantage of the fluid-based methods is the precise fluid manipulation. Ultimately, there is not a single ideal method of NSA reduction for all sensors. However, we suggest the optimal method to be chosen based on several factors: (1) Integration, (2) Multiplexibility, and (3) Tunability. First, the method of NSA reduction should be easily incorporated with the existing sensor structure. Note that many of the active removal methods are designed for microfluidic devices [10] while the passive methods could be applied to most surface-based sensors. Second, the method ideally can be applied to different sensing areas, since most sensors have or strive to have multiplexibility. Third, the method should be able to be adjusted for different experimental conditions, i.e., analyte/receptor combinations or device size. Other factors like ease of operation, and cost will play a role too.

When comparing biosensor performance, it is important to recognize the sensitivity depends on the analyte, recognition molecule, and surface immobilization method used [85]. The non-specific adsorption is highly dependent on all of those things, and repeatable results are of course necessary for successful demonstration of an NSA reduction method. Also, the authors point out one key limitation to measuring the efficiency of NSA removal methods is how researchers evaluate their devices’ anti-fouling ability. The most common method for evaluating adsorption is to compare the residual material left on the device after a typical use [105]. While this is direct and simple, it ignores transient adsorption. Hawkins et al. present a solution to this issue, developing a microfluidic device for transient monitoring of adsorption [105]. 

## 5. Conclusions and Future Trends

Non-specific adsorption is a critical problem in biosensing. While various methods exist to reconcile the problem, none of them are sufficient to create an ideal biosensor. In this review, we discussed the various passive and active methods of NSA reduction for biosensors. There is a trend present in the past decade of transitioning from surface chemistry NSA prevention to actively removing using transducer-based or fluid-based methods. A comprehensive table summarizing the reported methods of active NSA removal in biosensing and criteria for choosing a method of NSA reduction was given. Despite the extensive published literature, several important areas remain relatively unexplored. The first is investigating the use of various bioreceptor–analyte combinations in conjunction with transducer-based active NSA methods. The second is the ability of the NSA reduction method to not only limit NSA, but also improve specific binding, which a few studies claim. The last is combining active and passive NSA reduction methods in hopes of achieving further improved sensitivity. The research on methods to combat non-specific adsorption will likely continue to grow with the ever-expanding field of biosensor development and the increase in miniaturization technologies. With the shift from passive methods to active removal methods, the ability to use the more complex methods of NSA removal for a multitude of sensor types should be explored further. 

## Figures and Tables

**Table 1:** Summary of Active NSA Removal Methods.

Category	Type	Analyte	Reference
Transducer-based			
Electromechanical	Hypersonic Resonance	Cy3 labeled human IgG antigen	[9]
	ac-EHD	PSA [10]; IgG [10]; HER2 [10,73,74]; HER2 & PSA exosomes [75]; MCF7 & T-47D cells [76]	[10,73,74,75,76]
	Resonant Cantilever Vibration	BSA	[44,77]
Acoustic Wave	Surface	None (simulated) [62,78] IgG antigen [79]	[62,78,79]
	Orthogonal Surface	None (simulated) [22]	[22,80]
	Longitudinal	Biotinylated GNP-GSC	[81]
	Piezoelectric	Avidin	[38]
	Bulk	Proteins [82] None (simulated) [83]	[82,83]
Fluid-based			
	Hydrodynamic	HER2	[84]
